# Evaluating patient characteristics and trends of avoidable emergency department visits: Informing community health services to reduce emergency department utilization

**DOI:** 10.1177/13558196251358761

**Published:** 2025-07-09

**Authors:** Ryan P Strum, Andrew P Costa, Brent McLeod, Ravi Sivakumaran, Shawn Mondoux

**Affiliations:** 1 Research Institute of St Joe’s Hamilton, St Joseph’s Healthcare Hamilton, Hamilton, Canada; 2 Department of Health Research Methods, Evidence and Impact, 3710 McMaster University, Hamilton, Canada; 3 Hamilton Paramedic Services, Hamilton, ON, Canada; 4 Health Information Management Department, St Joseph’s Healthcare Hamilton, Hamilton, ON, Canada; 5 Department of Medicine, Division of Emergency Medicine, 3710 McMaster University, Hamilton, ON, Canada; 6 Department of Emergency Medicine, St Joseph’s Healthcare Hamilton, Hamilton, ON, Canada

**Keywords:** emergency department, health services, avoidable visit

## Abstract

**Background:**

There is a growing debate on whether avoidable emergency department (ED) visits, those involving health issues that could have been managed in community settings, represent a significant workload for the department. Until recently an ED physician-validated measure of avoidable visits has not been available, hindering our understanding of these patients, services rendered in the ED and the nature of their conditions. We examined patient characteristics of ED visits retrospectively classified as avoidable and potentially avoidable at a Canadian academic hospital.

**Methods:**

We conducted a retrospective cohort study using administrative ED data from an academic hospital in Hamilton, Canada from April 1, 2018 to August 31, 2023. We categorized all ED visits as avoidable, potentially avoidable, and not avoidable using the Emergency Department Avoidability Classification (EDAC). For each class, we analyzed patient characteristics and the top five physician interventions and diagnoses. We applied linear regression, locally weighted scatterplot smoothing (LOWESS) regression, and statistical process methods to examine monthly trends in avoidable and potentially avoidable visits. Additionally, we reported annual totals and length of stay for patients transported to the ED by paramedics.

**Results:**

Overall, 58,528 (29.0%) of 201,741 ED visits were classified as either avoidable (11,302; 5.6%) or potentially avoidable (47,226; 23.4%). These patients were predominantly young-to-middle aged, with average visit durations of 3 hours 33 minutes (avoidable) and 4 hours 26 minutes (potentially avoidable). Their primary interventions were predominantly diagnostic imaging, skin repairs and mental health assessments. The proportion of ED visits in the study period that were avoidable increased from 2.1% to 7.7% and potentially avoidable from 18.2% to 21.2%. Approximately one-in-five paramedic transported patients were classified as having either an avoidable or potentially avoidable ED visit. Transported patients had an average length of stay of 4 hours 22 minutes for avoidable visits and 4 hours 35 minutes for potentially avoidable visits.

**Conclusions:**

A notable rise in the proportion of ED visits that could have been managed in non-ED settings was observed. Providing community clinicians with resources and capacity to manage and refer patients for diagnostic imaging, skin repairs and mental health assessments may reduce avoidable ED attendance. Further exploration of avoidable ED visits transported by paramedics could support refining ED diversion care models. Hospitals and health service policymakers could benefit from similar analyses using validated measures to identify care gaps that inform the development of new health services and models tailored to the specific needs of their communities.

## Background

Canadian and international emergency departments (EDs) are confronting a significant challenge in providing care for escalating patient volumes.^1,2^ Difficulties in accessing primary care and limited health literacy underscore the necessity for innovative solutions to mitigate ED overutilization.^3–6^ There is an urgent need to explore alternative health care strategies to manage avoidable ED visits, which contribute to capacity strain. In Canada, EDs are striving to enhance health care system resilience by shifting care modalities to community-based settings for patients with low acuity and chronic medical conditions, aiming to reduce ED volumes and alleviate pressure on hospital resources.^7^

Avoidable ED visits occur when patients attend the ED for conditions that could have been appropriately managed and effectively treated in a non-ED health care setting.^8,9^ These patients often attend the ED for immediate consultation, diagnostic testing and medication administration to manage non-urgent conditions.^10^ Alternative non-ED settings that could accommodate such cases include urgent care centres, family practice clinics, telehealth services, and mental health crisis centres. Avoidable ED visits place a substantial burden on the health system by depleting scarce ED resources, increasing ED staff workload, prolonging patient wait times, and disrupting care continuity post-discharge.^11–14^

Addressing avoidable ED visits is an area of interest for both the research and health policy communities. Despite this focus, there remains a substantial gap in our understanding of the demographics of these patients, the specific services they receive in the ED, the conditions prompting their ED visits, and the proportion transported by paramedics. Previous research has reported widely varying estimates of avoidable ED visits, ranging from 2% to 90%, that prevent drawing any meaningful conclusions about avoidable visits.^9,15,16^ Conceptually, increasing equivalent services in the community that are sought during avoidable ED visits could potentially reduce avoidable ED attendance. However, the specific services that could fulfill this role are unknown or lack validation. From a pragmatic perspective, paramedics represent a sensible care model to benefit from a better understanding of avoidable ED visits, which may inform more targeted patient inclusion when redirecting patients from the ED to alternative care settings.^17^

Our primary objective was to identify the characteristics of avoidable and potentially avoidable ED visits at an academic hospital in Canada. Our secondary objective was to examine temporal trends of avoidable or potentially avoidable ED visits, including paramedic transports.

## Methods

### Study design

We conducted a retrospective cohort study using secondary administrative ED records from an academic hospital in Hamilton, Ontario, Canada. We adhered to the STrengthening the Reporting of OBservational studies in Epidemiology (STROBE) statement for the reporting of results (Table S1).^18^

### Population

All patients triaged in the ED of the academic hospital between April 1, 2018, and August 31, 2023 were eligible for inclusion. ED visits missing the variables needed to categorize their avoidable class were excluded (age, triage acuity, physician intervention, physician consult in the ED, ED outcome). Records before 2018 were not accessible, as 2018 was the first full year the ED recording system transitioned from paper-based to fully electronic. At the academic hospital, the ED was primarily staffed by ED physicians and nurses, with additional support from allied health professionals, including respiratory therapists and radiology technologists, as well as unit clerks and porters. Upon arrival, each patient had to undergo triage before officially registering as an ED patient, a process facilitated by an ED nurse to assess urgency and prioritize care accordingly.

### Variables and measurement

Our study used de-identified individual-level patient data measured and recorded at the time of ED visit. We selected patient characteristics for analyses based on prior literature, clinical judgement and data availability.^19^ Triage acuity was assigned by the ED triage nurse following ED registration using the Canadian Triage and Acuity Scale (CTAS). CTAS is an ordinal scale that ranges from one to five; one indicating the most severe (resuscitation) and five the least severe (non-urgent).^20^ Diagnostic categories were recorded using the Canadian Emergency Department Diagnostic Shortlist (CED-DxS).^21^ Primary physician interventions were recorded using the Canadian Classification of Health Interventions (CCI).^22^ Mode of transport to the ED were categorized as arriving by paramedics/ambulance or none (i.e., walk-in). The most responsible provider refers to the clinician who had primary responsibility for managing care at the time of ED visit. An ED consultation refers to an ED visit in which an inpatient unit physician consults with the ED physician regarding patient care in the ED.

ED visits were classified into three categories using the EDAC, a retrospective ED classifier of avoidable ED visits with established validity.^23^ Although several methodologies exist for classifying avoidable ED visits in retrospective data, such as CTAS, ambulatory care-sensitive conditions, and family practice-sensitive conditions, literature has shown that their estimates lack validity.^9^ This limitation underscores our rationale for using a validated classifier, the EDAC. Each ED visit was categorized as either avoidable, potentially avoidable or not avoidable.^23^ Avoidable ED visits could have been managed in non-ED care, potentially avoidable visits could have likely been managed in non-ED care, and non-avoidable visits could not have been managed outside of the ED.^23^ To meet the classification criteria for avoidable or potentially avoidable, patients had to be between 18 and 70 years old, discharged from the ED without admission, have no specialist physician consultation, receive a primary care-like intervention as their main physician intervention, and be triaged with a non-emergent acuity level, classified as less urgent (CTAS 4) or non-urgent (CTAS 5) for avoidable visits and urgent (CTAS 3) for potentially avoidable visits.^23^ Potentially avoidable visits have been shown to closely associated with avoidable ED visits, though with reduced classification performance.^23^ As a result, they represent cases that are likely suitable for community-based care.^23^

### Data source

Data were extracted from the academic hospital's central data repository, an ED incidence database that collects all patient care reports from all ED visits. All data were extracted by analysts of the Health Information Management Department (HIM) and de-identified at the academic hospital prior to analysis.

### Statistical analysis

We reported descriptive statistics of patient characteristics for each class of ED visit using measures of central tendency (frequencies, proportions). Excluded ED visits were stated directly with their rationale in the cohort creation. Additionally missing data were reported for each variable separately. Statistical differences in characteristics amongst the three classes were assessed using chi-squared tests, with a significance determined at a *p*-value threshold of 0.05. We used a time series figure to illustrate changes of combined avoidable and potentially avoidable ED visits over the study period, incorporating a linear regression line. Model performance was reported using the R-squared value and root mean squared error (RMSE) to assess fit of the trend. To account for the COVID-19 pandemic timeframe where ED utilization patterns had reduced, we applied locally weighted Scatterplot Smoothing (LOWESS) regression to the time series with 95% confidence intervals and reported its performance using R-squared and RMSE. The COVID-19 timeframe was considered February 1, 2020 to April 1, 2021.^24^ We reported the top five primary ED physician interventions and ED diagnoses for each class. Variations in temporal trends were analyzed using a statistical process control (SPC) analysis of the proportion of ED visits classified as avoidable and potentially avoidable. We computed the proportion for each month, the mean proportion for the study period (pbar), and three standard deviations from the overall proportion constituting the upper control limit (UCL) and lower control limit (LCL). Lastly, temporal changes of paramedic transported patients classified as having either avoidable or potentially avoidable ED visit were analyzed. Data were managed and analyzed in R statistical software (v.3.6).

### Ethical approval

Our study was approved by the Hamilton Integrated Research Ethics Board (HiREB), review reference number 2023-16838.

## Results

**Figure 1.**
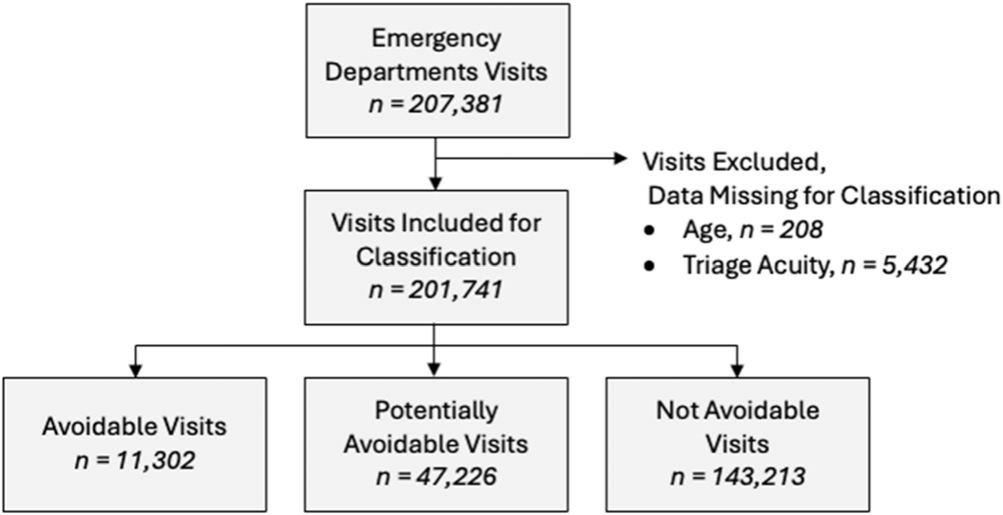
Cohort flow diagram.

Our study included 201,741 ED visits (97.3%) of the eligible 207,381 visits from the academic hospital. Figure 1 illustrates the cohort creation. Visits were excluded if they had missing age or triage acuity, given these components are necessary for the proper classification of ED visits using the EDAC criteria.

Table 1 shows the characteristics of all included ED visits. Overall, 58,528 ED visits (29.0%) were classified as either avoidable (11,302; 5.6%) or potentially avoidable (47,226; 23.4%). These ED visits had the highest proportion of patients aged 18 to 39 (52.9%; 48.3% vs 30.8%), attended the ED by walk-in (85.4%; 79.4% vs 64.5%) and were managed by an emergency medicine physician (99.7%; 99.7% vs 58.6%). Not avoidable ED visits presented with either emergent (45.8% vs 0% avoidable; 0% potentially avoidable) or urgent (47.1% vs 0.0% avoidable; 100% potentially avoidable) acuity levels, and nearly one-third resulted in hospital admission (31.6% vs 0% avoidable; 0% potentially avoidable).

**Table 1.** Characteristics of emergency department visits classified by avoidability from April 1, 2018 to June 30, 2023 at an academic hospital in Hamilton, Canada.

Characteristic	Emergency department avoidability class, *n* (%)			*p*-value
	Avoidable	Potentially avoidable	Not avoidable	
ED visits, total	11,302 (5.6)	47,226 (23.4)	143,213 (71.0)	
Sex				<0.05
Male	5,990 (53.0)	22,128 (46.9)	65,778 (45.9)	
Female	5,305 (46.9)	25,082 (53.1)	77,366 (54.0)	
Other	6 (0.1)	14 (0.0)	60 (0.1)	
Unknown	1 (0.0)	2 (0.0)	9 (0.0)	
Age, years				
0 – 17	0 (0.0)	0 (0.0)	2,505 (1.7)	
18 – 39	5,984 (52.9)	22,831 (48.3)	44,109 (30.8)	
40 – 59	3,788 (33.5)	16,335 (34.6)	33,781 (23.6)	
60 – 74	1,530 (13.5)	8,060 (17.1)	29,605 (20.7)	
75 – 106	0 (0.0)	0 (0.0)	33,213 (23.2)	
ED arrival mode				<0.05
Ambulance	1,654 (14.6)	9,740 (20.6)	50,857 (35.5)	
Walk-in	9,648 (85.4)	37,486 (79.4)	92,356 (64.5)	
Triage, CTAS				
1 – Resuscitation	0 (0.0)	0 (0.0)	2,137 (1.5)	
2 – Emergent	0 (0.0)	0 (0.0)	65,574 (45.8)	
3 – Urgent	0 (0.0)	47,227 (100.0)	67,431 (47.1)	
4 – Less urgent	8,334 (73.7)	0 (0.0)	6,083 (4.2)	
5 – Non-urgent	2,969 (26.3)	0 (0.0)	1,988 (1.4)	
Triage time of day				<0.05
Morning (0600-1159)	2,567 (22.7)	11,444 (24.2)	3,4936 (24.4)	
Afternoon (1200-1759)	3,407 (30.2)	16,954 (35.9)	55,683 (38.9)	
Evening (1800-2359)	3,648 (32.3)	13,135 (27.8)	37,738 (26.4)	
Overnight (0000-0559)	1,680 (14.9)	5,693 (12.1)	14,798 (10.3)	
Unknown	0 (0.0)	0 (0.0)	59 (0.0)	
Day of the week				<0.05
Weekday (Mon to Fri)	8,246 (73.0)	34,540 (73.1)	106,137 (74.1)	
Weekend (Sat and Sun)	3,056 (27.0)	12,686 (26.9)	37,076 (25.9)	
Most responsible provider				<0.05
Emergency medicine	11,273 (99.7)	47,064 (99.7)	83,883 (58.6)	
Critical care medicine	0 (0.0)	0 (0.0)	1,382 (1.0)	
General internal medicine	0 (0.0)	0 (0.0)	5,806 (4.1)	
Internal medicine	0 (0.0)	0 (0.0)	20,281 (14.2)	
Nephrology	0 (0.0)	0 (0.0)	3,580 (2.5)	
Orthopedic surgery	0 (0.0)	0 (0.0)	2,069 (1.4)	
Psychiatry	3 (0.0)	22 (0.0)	19,193 (13.4)	
Urology	0 (0.0)	0 (0.0)	1,542 (1.1)	
Other	26 (0.3)	140 (0.3)	5,477 (3.8)	
ED visit outcome				
Discharged	11,302 (100.0)	47,226 (100.0)	93,862 (65.5)	
Admitted to receiving hospital	0 (0.0)	0 (0.0)	45,304 (31.6)	
Transferred to other acute center	0 (0.0)	0 (0.0)	3,435 (2.4)	
Died in emergency department	0 (0.0)	0 (0.0)	349 (0.3)	
Other	0 (0.0)	0 (0.0)	263 (0.2)	
Total time spent in ED^a^, mean	3:33	4:26	10:50	

*Note.* CTAS = Canadian Triage and Acuity Scale.

^a^Time from triage to left ED, shown as h:mm.

**Figure 2.**
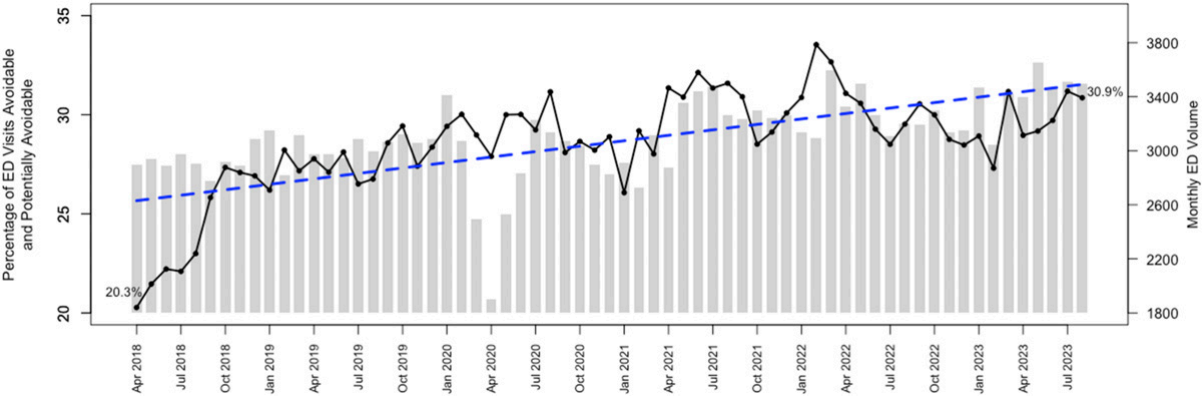
Trend of the combined ED visits classified as avoidable and potentially avoidable in a Canadian academic hospital.

Figure 2 illustrates the percentage of ED visits per month classified as either avoidable or potentially avoidable, and total ED visits per month. A positive trend line is shown over the analyzed timeframe, with a 10.6% change from April 2018 (20.3%) to August 2023 (30.9%). The LOWESS regression is shown in Table S2.

Table 2 shows the top five most frequently recorded primary physician interventions and diagnoses. The top five most frequently recorded primary physician interventions comprised 47.5% of all avoidable ED visits, and 57.1% of all potentially avoidable ED visits. The top five most frequently recorded diagnoses comprised 24.5% of all avoidable ED visits, and 27.4% of all potentially avoidable ED visits. Of the top five interventions for avoidable ED visits, three related to skin repair, and the remaining associated with X-ray imaging. All top five potentially avoidable interventions consisted of diagnostic imaging (X-ray, ultrasound) and mental health assessments. Not avoidable visits predominantly involved imaging procedures (computerized tomography, X-ray) and mental health assessments. All three classes exhibited very similar top 5 diagnoses, with three conditions being included in all classes: abdominal and pelvic pain, pain in throat and chest, and reaction to severe stress.

**Table 2.** List of top five physician primary interventions and patient diagnoses of avoidable, potentially avoidable and not avoidable ED visits.

Rank	Emergency department avoidability class		
	Avoidable	Potentially avoidable	Not avoidable
Primary physician intervention, CCI			
1	X-ray, thoracic cavity without contrast (with or without fluoroscopy)	X-ray, thoracic cavity without contrast (with or without fluoroscopy)	X-ray, thoracic cavity without contrast (with or without fluoroscopy)
2	Assessment mental health capacity	Ultrasound, abdominal cavity alone	Computerized tomography, head without contrast
3	X-ray foot without contrast	Assessment, mental health and addictions for other reason	Assessment, mental health and addictions for capacity for harm (to self or others)
4	X-ray ankle without contrast	Assessment, mental health and addictions for capacity for harm (to self or others)	Computerized tomography, abdominal cavity without contrast
5	Repair, skin of hand using apposition technique [suture]	X-ray, ankle joint without contrast (e.g. plain film) (with or without fluoroscopy)	Assessment, mental health and addictions for other reason
Total^a^	5,364 / 11,302 (47.5)	26,988 / 47,226 (57.1)	79,469 / 143,213 (55.5)
Emergency department diagnostic category, ICD-10-CA			
1	Open wound of wrist and hand	Abdominal and pelvic pain	Pain in throat and chest
2	Abdominal and pelvic pain	Reaction to severe stress	Abdominal and pelvic pain
3	Pain in throat and chest	Pain in throat and chest	Reaction to severe stress
4	Superficial injury of ankle and foot	Other anxiety disorder	Other chronic obstructive pulmonary disease
5	Reaction to severe stress	Open wound of wrist and hand	Depressive episode
Total^a^	2,765 / 11,302 (24.5)	12,956 / 47,226 (27.4)	22,803 / 143,213 (15.9)

*Note.* CCI = Canadian Classification of Health Interventions, ICD-10-CA = International Statistical Classification of Diseases and Related Health Problems, Tenth Revision, Canada.

^a^Total of the top five in each EDAC class, shown as *n* (%).

Over the study period, the proportion of avoidable and potentially avoidable ED visits both increased by 6%. Table S3 shows a p-chart of the proportion of avoidable and potentially avoidable ED visits in the study period. For avoidable visits, a u-shaped trend was observed, with a large increase at the beginning of the study period (steep incline from below the LCL to above the UCL), and a progressive reduction during the COVID-19 pandemic. After the pandemic, points fluctuated around the pbar line (0.06), with the final 18 months showing a positive trend, ending with four points exceeding the UCL. For potentially avoidable visits, a positive trend was observed in the early years, followed by fluctuations during and following the COVID-19 pandemic that included 3 points above the UCL. The general trend at the conclusion of the period resided near the pbar line (0.23), fluctuating within the LCL and UCL.

Table 3 shows a subgroup analysis of paramedic transported ED visits classified as avoidable and potentially avoidable. Throughout the study period, the proportion of transports either avoidable or potentially remained relatively consistent, ranging from 17% to 19%. Avoidable visits comprised 2.7% of the patients transported, and potentially avoidable visits were 15.6%. On average, transported patients who had avoidable and potentially avoidable ED visits spent nearly four and a half hours in the ED for their visit (4:22, 4:25 respectfully).

**Table 3.** Paramedic transported ED visits that were retrospectively classified as avoidable and potentially avoidable.

	Overall	2018	2019	2020	2021	2022	2023	Time in ED, mean^a^
Paramedic transports, total	62,251	8,583	11,335	11,076	11,673	11,626	7,958	-
Avoidable, *n* (%)	1,654 (2.7)	221 (2.6)	329 (2.9)	301 (2.7)	295 (2.5)	301 (2.6)	207 (2.6)	4:22
Potentially avoidable, *n* (%)	9,740 (15.6)	1,278 (14.9)	1,650 (14.6)	1,784 (16.1)	1,931 (16.5)	1,868 (16.1)	1,229 (15.4)	4:35
Either avoidable or potentially avoidable, *n* (%)	11,394 (18.3)	1,499 (17.5)	1,979 (17.5)	2,085 (18.8)	2,226 (19.1)	2,169 (18.7)	1,436 (18.0)	-
ED visits, total	201,741	26,262	36,396	34,448	38,192	38,989	27,454	-
Proportion of ED visits								
Transported by paramedics, %	30.9	32.7	31.1	32.2	30.6	29.8	29.0	-

^a^Shown in hour:minutes.

## Discussion

Overall, 29% of ED visits were classified as either avoidable (6%) or potentially avoidable (23%), with an increasing trend over the study period in both groups. These patients primarily received interventions of diagnostic imaging, mental health assessments and skin repairs. The larger proportion of potentially avoidable ED visits likely reflects the difficulty in classifying an ED visit as avoidable due to inherent limitations of using administrative data, which may not capture the full clinical complexity of patient presentations. Avoidable and potentially avoidable ED visits consistently accounted for nearly one in four paramedic transported patients. Expanding community-based care models capable of delivering these services may help reduce increasing ED utilization.

Our findings align with existing literature estimating the proportion of avoidable and potentially avoidable ED visits using the EDAC.^15^ Patients of avoidable ED visits in our study were predominantly young-to-middle aged adults, an outcome consistent with prior research.^9,25,26^ Although several methodologies exist for classifying avoidable ED visits, the lack of validity evidence has made their application unreliable in ED data, leading the research community to question their usefulness.^9,27,28^ In contrast, the EDAC has demonstrated criterion validity, supporting its use as a more robust classification tool.^23^ Our findings regarding identification of the most frequently recorded primary physician interventions, and quantification of paramedic transported patients have not been extensively described in the literature.

Our results call attention for the need to explore strengthening subacute, primary, community and specialized care services that could mitigate avoidable ED overuse. These services should prioritize providing access to diagnostic imaging, capacity for community clinicians to refer patients to imaging, interventions for minor skin injury repair and assessment for mental health needs. Specifically, targeting regions where individuals have high ED utilization that would later be categorized as avoidable visits may be particularly effective. Policymakers can use this evidence to support investments in urgent care centres, virtual care, and expanded primary care access, reducing reliance on EDs for low-acuity conditions. By quantifying avoidable and potentially avoidable ED visits, this research informs policy decisions on workforce planning and resource distribution, ensuring that emergency care capacity is prioritized for acute emergencies. Our analysis did not explore the motivations behind individuals seeking ED care for avoidable conditions, nor did it assess the accessibility of community resources that might have been utilized. Further research and understanding are needed to uncover the upstream factors influencing individual decisions to utilize ED services instead of community healthcare.

We found similar diagnostic categories across avoidability categories, indicating the limitations of using diagnostics information alone to differentiate avoidable ED visits. These findings provide further evidence that leveraging a classification tool with known evidence of validity a prudent strategy to retrospectively identify avoidable ED visits, as opposed to unvalidated methods reliant on diagnostics alone.

Given the consistent proportion of paramedic transports that could have been managed in non-ED settings, and considering the timing of Canada’s health policymakers authorizing new care models for paramedics to transport patients to non-ED care centres, further exploration of this subgroup is warranted to inform community identification of patients suitable for ED redirection.^17^ Expanding the paramedic practice scope in primary care modalities has shown to be an effective model in some provinces of Canada, leading to decreased ED visits when providing treatment in home and making referrals on site.^29^ Canadian provinces that have not integrated expanded paramedic care models could likely benefit from adopting a similar approach to alleviate mounting pressures on their EDs and healthcare systems.

## Limitations

Underlying factors influencing avoidable ED visits could not be explored as we only analyzed ED visit data. There is an element of missing data inherent to retrospective cohort studies, though the proportion of missing data in our cohort was consistent with their normal completion as fields in secondary administrative ED data.^23,30^ Our analysis was based on records from a single academic hospital. Expanding our analysis to multiple hospital sites could have strengthened the robustness of our results.

## Conclusion

The proportion of ED visits that could have been managed in non-ED settings has risen; albeit the proportion of avoidable visits remains small. Patients with avoidable and potentially avoidable visits primarily seek the ED for diagnostic imaging, skin repairs and mental health assessments. Providing community clinicians with the capacity to offer these services and enhancing patient access to them may reduce avoidable ED visitation. Potentially avoidable ED visits indicate greater uncertainty, but may also be amendable to reduction through such measures. Exploring paramedic transported patients who had avoidable ED visits will be important to specify criteria for redirection care models to non-ED settings.

## Supplemental Material

Supplemental Material - Evaluating patient characteristics and trends of avoidable emergency department visits: Informing community health services to reduce emergency department utilization Supplemental Material for Evaluating patient characteristics and trends of avoidable emergency department visits: Informing community health services to reduce emergency department utilization by Ryan P Strum, Andrew P Costa, Brent McLeod, Ravi Sivakumaran, Shawn Mondoux in Journal of Health Services Research & Policy.

## Data Availability

The datasets generated during and/or analyzed during the current study are not publicly available due personal health information restrictions in Canada but are available from the corresponding author on reasonable request.*
